# An international multicenter retrospective analysis of repeated anti-ENA testing in ANA-associated rheumatic diseases, a data-driven proposal to increase testing efficacy

**DOI:** 10.1016/j.jtauto.2025.100298

**Published:** 2025-06-25

**Authors:** Wim H.M. Vroemen, Maria Infantino, Mariangela Manfredi, Joyce J.B.C. van Beers, Carolien Bonroy, Jan G.M.C. Damoiseaux

**Affiliations:** a Central Diagnostic Laboratory, Maastricht University Medical Center+, Maastricht, The Netherlands; b Immunology and Allergology Laboratory, S. Giovanni di Dio Hospital, Florence, Italy; c Department of Laboratory Medicine, Ghent University Hospital, Ghent, Belgium; d Department of Diagnostic Science, Ghent University, Ghent, Belgium

**Keywords:** ANA-Associated rheumatic diseases, Extractable nuclear antigen antibodies, Laboratory algorithms

## Abstract

**Background:**

Autoantibodies detection in ANA-associated rheumatic diseases (AARD) is not only used for diagnostic and classification purposes, but also for monitoring. In case of AARD it is questioned if repeated anti-ENA testing is of any substantial value. In this international multicenter retrospective study, repeated anti-ENA testing according to local AARD testing algorithms was investigated.

**Methods:**

Anti-ENA results (anti-SSA60, -Ro52, -SSB, -Scl-70, -CENP-B, -RNP, -Sm) over a 6 to 10-year period were extracted from the laboratory information systems of three participating centers. Time between repeated testing was determined and concordance analysis was performed.

**Results:**

The study included 28557 anti-ENA requests from 19388 patients (72 % female). In 15227 patients (78.5 %) only one anti-ENA test was performed (79.9 % negative), while 4161 patients (21.5 %) underwent multiple (median [interquartile range (IQR)]; 2 [2–4]) tests with a maximum of 31 tests. The median [IQR] time interval between anti-ENA testing for the total cohort was 364 [195–539] days. Concordance analysis demonstrated that repeated anti-ENA test results did not show any change in 3583 patients (86.1 %). Additional autoantibodies were observed in 243 patients (5.8 %). In 76 (1.8 %) patients a positive anti-ENA test was obtained after an initial negative anti-ENA test result, while in 167 (4.0 %) patients additional autoantibodies were detected after an initial positive anti-ENA result.

**Conclusions:**

Repeated anti-ENA testing with a median time interval of about one year is common independently of local laboratory testing algorithms, but showed a limited added value since only 1.8 % of the patients have demonstrated a positive anti-ENA test after an initial negative anti-ENA test. These data at least suggest that repeated anti-ENA tests should be discouraged and only be instigated by a change in clinical manifestations.

## 1 Introduction

Antibodies against extractable nuclear antigens (ENA) are a characteristic feature of antinuclear antibody (ANA)-associated rheumatic diseases (AARD) such as systemic lupus erythematosus (SLE), systemic sclerosis (SSc), mixed connective tissue disease (MCTD) and Sjögren syndrome (SS) [[1], [2], [3]]. Many of the anti-ENA antibodies are specific for the distinct AARD and are therefore included in the classification criteria of the respective disease. However, testing for autoantibodies is not only used for diagnostic or classification purposes, but is also frequently used for the follow-up of patients. Monitoring autoantibody levels can be particularly important for assessing treatment efficacy in various autoimmune conditions, such as celiac disease [4] and Goodpasture's disease [5], where changes in antibody titers of anti-tissue transglutaminase and anti-glomerular basement membrane, respectively, may reflect response to therapy. Additionally, measuring autoantibody levels can aid in predicting disease flares or relapses, as demonstrated in systemic lupus erythematosus (SLE) [6] and anti-neutrophil cytoplasmic antibody (ANCA)-associated vasculitis [7,8], enabling more timely clinical interventions. However, in the context of ANA-associated rheumatic diseases (AARD), the clinical utility and added value of repeated anti-ENA antibody testing during patient follow-up remain uncertain and subject to ongoing debate.

Until recently, repeated anti-ENA testing has only been examined in a small population of patients with SLE showing low variability over time [9]. More recently, a larger retrospective study in a tertiary hospital has shown that repeated anti-ENA testing is common in routine clinical practice, though a change in anti-ENA results was rarely observed with a very low diagnostic power [10]. Hence, it was recommended that repeated anti-ENA testing (as well as ANA [11]) should be avoided unless the pre-test probability of AARD is altered by a change in clinical manifestations. However, this recommendation was based on a single center study and it is important to note that the anti-ENA testing landscape has changed dramatically during the last decades. The methods for detecting anti-ENA specificities have greatly changed over time, from singleplex to multiplex platforms enabling the simultaneous detection of several antibodies. As a result of this, today we have numerous different assays based on diverse methodologies [[12], [13], [14]] from multiple manufacturers with relevant differences in their performance characteristics [15]. Additionally, to date there is no universal guideline on laboratory algorithms for evaluating AARD for classification, diagnostic or monitoring purposes. Thus far, recommendations have been made only to facilitate the design of local algorithms for detecting anti-nuclear antibodies [15]. As the methods and algorithms influence the process of harmonizing results, proper interpretation requires that clinicians are aware of the way anti-ENA antibodies are detected and reported [16,17]. Indeed, to fully assess if repeated anti-ENA testing is of added value or not, a broader spectrum of assays and algorithms should be investigated.

In this international multi-center study, we aimed to investigate the real-world practice of repeated anti-ENA antibody testing in patients with AARD. The study was conducted across three distinct centers, each employing different anti-ENA testing methodologies and laboratory algorithms, reflecting the diversity in clinical and laboratory practices worldwide. By evaluating these varied approaches, the study seeks to provide a broader understanding of the clinical utility, consistency, and potential added value of repeated anti-ENA testing in routine patient follow-up, thereby addressing current gaps and variability in testing strategies across different healthcare settings.

## 2 Materials and methods

### 2.1 Study design and laboratory algorithms

**Fig. 1:**
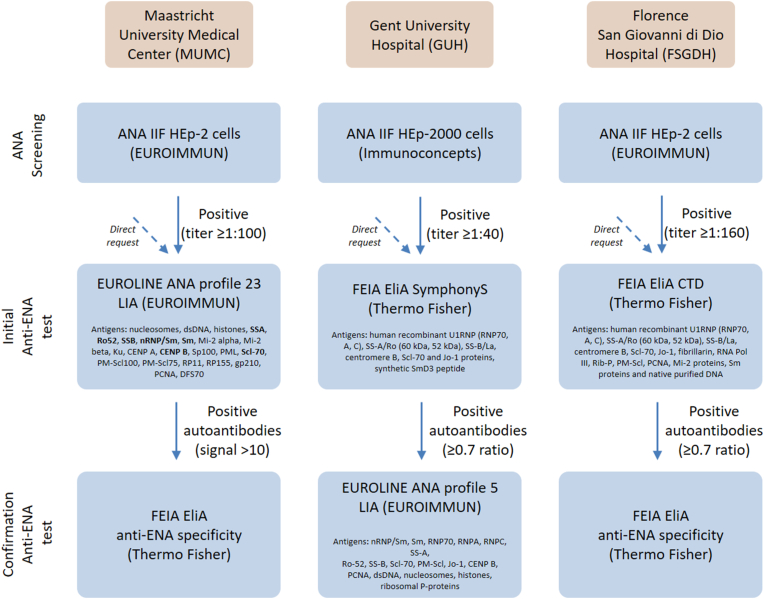
Flowchart of diagnostic algorithm for ANA/anti-ENA reflex testing according to the different protocols of the three participating centers; Maastricht University Medical Center (MUMC), Gent University Hospital (GUH) and Florence San Giovanni di Dio Hospital (FSGDH). ANA = antinuclear antibody; FEIA = fluorescent-enzyme immunoassay; IIF = indirect immunofluorescence; LIA = line immunoassay.

This is an observational retrospective multicenter study involving three different centers with their own specific ANA/ENA test algorithm (Fig. 1). Although often many more antibodies have been tested in the three participating centers, the investigated autoantibodies were limited to antibodies against SSA/Ro-60, Ro-52, SSB, Scl-70, CENP-B, RNP and Sm since these were the overlapping denominators.

#### 2.1.1 Maastricht University Medical Center (MUMC)

There are separate algorithms for patients with or without previous anti-ENA test results. For patients with an initial anti-ENA test request, an indirect immunofluorescence (IIF) assay on HEp-2 cells (EUROIMMUN, Lübeck, Germany) is conducted. If the HEp-2 IIF assay is negative, no further testing is performed, unless specifically requested. If the HEp-2 IIF is positive (titer ≥1:100), and depending on the HEp-2 IIF pattern (homogeneous, centromere, speckled, nucleolar), the EUROLINE ANA profile 23 (IgG) line immunoassay (LIA) is performed for anti-ENA detection (EUROIMMUN). Positive autoantibody results by LIA, as far as included in the current study, are subsequently confirmed with a monospecific EliA IgG test, a fluorescent-enzyme immunoassay (FEIA), on the Phadia 250 system (Thermo Fisher Scientific, Freiburg, Germany). The anti-ENA is considered positive if the confirmation by FEIA is positive.

For patients with previous anti-ENA test results, the LIA is conducted. When historically identified autoantibodies are detected, no further confirmation by FEIA is performed. New autoantibody specificities are confirmed with FEIA as described above. Additionally, if only ANA is requested in repeat analysis and the ANA pattern and titer is identical to the initial ANA result, no ENA testing (neither LIA nor FEIA) is performed.

#### 2.1.2 Ghent university hospital (GUH)

For patients with an initial anti-ENA test request, an IIF assay on HEp-2000 cells (Immunoconcepts, Sacramento, CA) is performed. If the HEp-2000 IIF is negative, no further testing is performed, unless specifically requested. If the HEp-2000 IIF is positive (titer ≥1:40), samples are further tested using the EliA SymphonyS on the Phadia 250 instrument (Thermo Fisher Scientific). Samples tested as borderline or positive on EliA Symphony (≥0.7 ratio) are tested on EUROLINE ANA profile 5 (IgG) LIA (EUROIMMUN). Anti-ENA positivity is defined using cut-off values as proposed by the manufacturer (positive: >10 arbitrary units [AU], borderline: 6–10 AU, negative: ≤6 AU). Anti-RNP is considered positive when native mRNP/Sm complex and at least one recombinant antigen (RNP70, RNPA, RNPC) is positive. If the EliA SymphonyS is negative, no further LIA testing is performed, unless specifically requested, or if the ANA pattern suggests an anti-ENA not fully covered by the EliA SymphonyS (e.g. AC-19/20).

#### 2.1.3 Florence S. Giovanni di Dio Hospital (FSGDH)

For patients with an anti-ENA request, an IIF on HEp-2 cells is performed (EUROIMMUN). If the HEp-2 cells IIF is positive (titer ≥1:160), the EliA CTD Screen on the Phadia 250 system is performed (Thermo Fisher Scientific). Samples borderline or positive on EliA CTD (≥0.7 ratio) were further analyzed by monospecific EliA IgG assays on the Phadia 250 system (Thermo Fisher Scientific). The ENA is considered positive if the confirmation with the monospecific EliA IgG assay is positive.

In addition, the CTD screen may also be directly requested by clinicians in support of a strong clinical suspicion, independently from the IIF test.

### 2.2 Data extraction and statistical analysis

The data extracted from the laboratory information systems are provided in pseudonymized form and included the following patient information: patient code, age (if available), sex, anti-ENA test results. Incomplete test results and external requests were excluded from the analysis. In patients with multiple anti-ENA results, the time period between testing was calculated and concordance analyses were performed. Concordance was defined as no change in anti-ENA specificity results across all anti-ENA tests for each patient.

Results are presented as median [interquartile range (IQR)] or mean ± standard deviation depending on Gaussian distribution. Because the laboratory algorithms, patient numbers, and studied timeframes vary among the three participating centers, solely descriptive statistics was used in which percentages between centers were compared rather than absolute counts (though available in the Tables). Figures were created using GraphPad Prism (version 5.04, La Jolla, CA) or R (4.4.1) using packages ggplot2 (3.5.1), ggalluvial (0.12.5) and dplyr (1.1.4).

### 2.3 Ethical approval

This study was conducted according to the Declaration of Helsinki. Ethical approval was not required as this study solely used retrospectively collected pseudonymized data collected by routine clinical care from the laboratory information systems of the three participating hospitals. Data transfer agreements were established when required by the participating center.

## 3 Results

### 3.1 Baseline patient characteristics

**Fig. 2:**
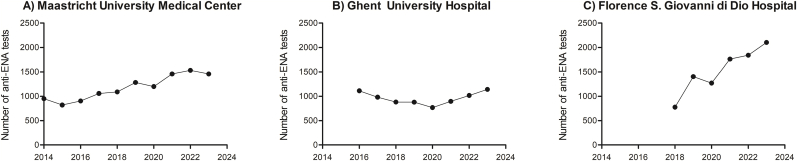
Number of anti-ENA analyses per year during the studied timeframe per participating center.

This study analysis has included 28557 anti-ENA results from 19388 patients of three different participating centers in a timeframe ranging from six to ten years (Table 1). Differences in timeframes between participating centers were due to changes in the local algorithm prior to the indicated timeframe. The number of anti-ENA analyses performed increased over time for MUMC and FSGDH, while declined for GUH from 2016 to 2020 and subsequently increased from 2020 to date (Fig. 2). Additionally, all centers had a decrease in anti-ENA tests in calendar year 2020.

**Table 1:** Baseline patient characteristics and anti-ENA test results.

	Total	Maastricht University Medical Center	Ghent University Hospital	Florence S. Giovanni di Dio Hospital
**Timeframe**	–	1-jan-2014 – 31-dec-2023	1-jan-2016 – 31-dec-2023	22-feb-2018 – 31-dec-2023
**Anti-ENA tests *n* (%)**				
Total	28557	11741	7667	9149
Negative	15857 (55.5 %)	10165 (86.6 %)	2396 (31.3 %)	3296 (36.0 %)
Borderline^a^	675 (2.4 %)	0 (0.0 %)	232 (3.0 %)	443 (4.8 %)
Positive	12025 (42.1 %)	1576 (13.4 %)	5039 (65.7 %)	5410 (59.1 %)
**Patients *n***	19388	9920	4140	5328
**Age∗∗ mean±SD**	–	54 ± 17	–	61 ± 17
**Female *n* (%)**	13896 (71.7 %)	6465 (65.2 %)	3140 (75.8 %)	4291 (80.5 %)
**Anti-ENA positive patients based on 1**st **sample *n* (%)**				
Total	5597 (28.9 %)	878 (8.9 %)	2005 (48.4 %)	2714 (50.9 %)
Female	4682 (83.7 %)	682 (77.7 %)	1630 (81.3 %)	2370 (87.3 %)

ENA = extractable nuclear antigen.

a Borderline was considered negative according to the manufacturer's instructions for use. ∗∗Age at the time of the first anti-ENA test result, if available.

There was a substantial difference in the percentage of positive anti-ENA tests results in total, especially between MUMC (13.4 %) when compared to GUH (65.7 %) and FSGDH (59.1 %). This difference between centers was also observed for anti-ENA positive patients based on the first sample; MUMC 8.9 %, GUH 48.4 % and FSGDH 50.9 % (Table 1). Female patients accounted for most anti-ENA tests in all centers (overall 71.7 %). Additionally, 83.7 % of all patients with a positive anti-ENA result based on the first sample were female (Table 1).

### 3.2 Anti-ENA antigen specificity test results

Based on the first sample, there were 8579 anti-ENA positive specificities for 5597 patients (Table 2). The most prevalent antibodies detected were against SSA60 (n = 2656, 31.0 %) and Ro52 (n = 2664, 31.1 %) followed by CENP-B (n = 1243, 14.5 %), SSB (n = 879, 10.2 %), RNP (n = 521, 6.1 %), Scl70 (n = 314, 3.7 %) and Sm (n = 302, 3.5 %). The prevalence of anti-ENA specificities were comparable among the three centers. In addition, single positive ENA specificity was observed in 3517 patients (62.8 %), 1287 (23.0 %) patients having two, 702 (12.5 %) three, 78 (1.4 %) four and 13 (0.2 %) patients having five or more positive anti-ENA specificities.

**Table 2:** Anti-ENA antigen specificity test results based on the patient's initial blood sample.

	Total	Maastricht University Medical Center	Ghent University Hospital	Florence S. Giovanni di Dio Hospital
**Positive anti-ENA specificity based on 1**st **sample *n* (%)**				
Total	8579	1416	3129	4034
SSA60/Ro60	2656 (31.0 %)	451 (31.9 %)	828 (26.5 %)	1377 (34.1 %)
Ro-52	2664 (31.1 %)	427 (30.2 %)	1076 (34.4 %)	1161 (28.8 %)
SSB	879 (10.2 %)	164 (11.6 %)	277 (8.9 %)	438 (10.9 %)
Scl-70	314 (3.7 %)	43 (3.0 %)	169 (5.4 %)	102 (2.5 %)
CENP-B	1243 (14.5 %)	180 (12.7 %)	551 (17.6 %)	512 (12.7 %)
RNP	521 (6.1 %)	113 (8.0 %)	156 (5.0 %)	252 (6.2 %)
Sm	302 (3.5 %)	38 (2.7 %)	72 (2.3 %)	192 (4.8 %)
**Number of positive anti-ENA specificities based on 1**st **sample *n* (%)**				
Total	5597	878	2005	2714
1	3517 (62.8 %)	513 (58.4 %)	1194 (59.6 %)	1810 (66.7 %)
2	1287 (23.0 %)	213 (24.3 %)	541 (27.0 %)	533 (19.6 %)
3	702 (12.5 %)	136 (15.5 %)	232 (11.6 %)	334 (12.3 %)
4	78 (1.4 %)	11 (1.3 %)	34 (1.7 %)	33 (1.2 %)
5	10 (0.2 %)	5 (0.6 %)	3 (0.1 %)	2 (0.1 %)
6	1 (0.0 %)	0 (0.0 %)	1 (0.0 %)	0 (0.0 %)
7	2 (0.0 %)	0 (0.0 %)	0 (0.0 %)	2 (0.1 %)

ENA = extractable nuclear antigen.

### 3.3 Single and repeated anti-ENA test results

In total, 15227 (78.5 %) patients underwent only one anti-ENA test (Table 3). Interestingly, MUMC had the most patients with only one measurement (89.1 %) and also the highest percentage of negative anti-ENA tests (93.8 %). In contrast, GUH (69.8 %) and FSGDH (65.7 %) had a lower number of patients with only one anti-ENA test as well as a lower negative anti-ENA frequency, 65.1 % and 57.1 %, respectively. The rate of a positive anti-ENA result among female patients (81.1 %) was greater than that of a negative result (65.7 %).

**Table 3:** Results of anti-ENA testing in patients with only one test performed during the study timeframe.

	Total	Maastricht University Medical Center	Ghent University Hospital	Florence S. Giovanni di Dio Hospital
**Patients with 1 anti-ENA test *n* (%)**				
Total	15227 (78.5 %)	8834 (89.1 %)	2891 (69.8 %)	3502 (65.7 %)
Negative	12171 (79.9 %)	8288 (93.8 %)	1883 (65.1 %)	2000 (57.1 %)
Positive	3056 (20.1 %)	546 (6.2 %)	1008 (34.9 %)	1502 (42.9 %)

ENA = extractable nuclear antigen.

**Fig. 3:**
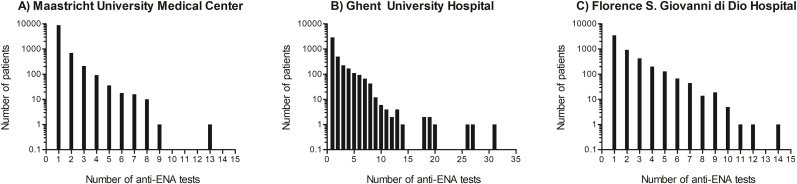
Number of anti-ENA analyses per patient per participating center within in the studied timeframe.

Out of the total number of patients, 4161 (21.5 %) patients had multiple anti-ENA tests (Fig. 3). The median [IQR] number of anti-ENA tests was 2 [[2], [3], [4]] with a maximum of 31. In contrast to MUMC (10.9 %), repeated testing was predominantly performed at FSGDH (34.3 %) and GUH (30.2 %). The median [IQR] time window between anti-ENA testing was 364 days [195−539] for all centers combined and differed between the participating centers (Table 4).

**Table 4:** Anti-ENA test results of patients with multiple anti-ENA tests.

	Total	Maastricht University Medical Center	Ghent University Hospital	Florence S. Giovanni di Dio Hospital
**Patients with ≥2 anti-ENA tests *n* (%)**	4161	1086 (10.9 %)	1249 (30.2 %)	1826 (34.3 %)
**Number of repeated anti-ENA tests (median [IQR])**	2 [[2], [3], [4]]	2 [2,3]	2 [[2], [3], [4]]	3 [[2], [3], [4], [5]]
**Days between anti-ENA testing (median [IQR])**	364 [195–539]	487 [239–943]	364 [210–469]	311 [179–465]
**Concordance analysis of patients with multiple anti-ENA test results *n* (%)**				
Stable positive	2040 (49.0 %)	261 (24.0 %)	768 (61.5 %)	1011 (55.4 %)
Stable negative	1543 (37.1 %)	737 (67.9 %)	220 (17.6 %)	589 (32.3 %)
Change positive	243 (5.8 %)	38 (3.5 %)	128 (10.2 %)	77 (4.2 %)
Change negative	335 (8.1 %)	50 (4.6 %)	133 (10.6 %)	152 (8.3 %)
**Anti-ENA autoantibody changes n (%)**				
Total	967	126	490	351
Additional	416 (43.0 %)	54 (42.9 %)	230 (46.9 %)	132 (37.6 %)
Fewer	551 (57.0 %)	72 (57.1 %)	260 (53.1 %)	219 (62.4 %)

ENA = extractable nuclear antigen, IQR = interquartile range.

### 3.4 Concordance analysis of repeated anti-ENA testing

**Fig. 4:**
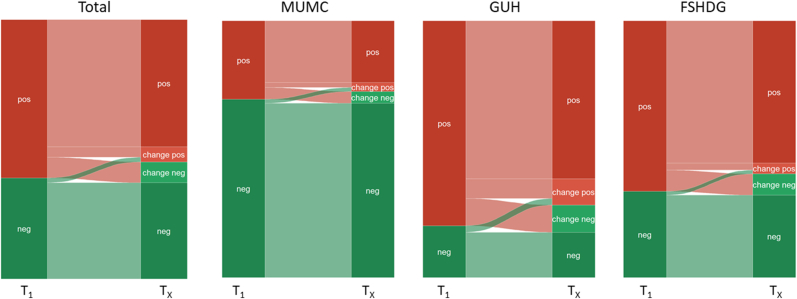
Concordance analysis, graphically showed with an alluvial plot, of patients with multiple anti-ENA tests (n = 4161) combined as well as per participating center. T_1_ represent the first anti-ENA test result, while T_X_ represents the first alteration, if applicable. Most patients had identical results (T_1_ pos and T_X_ pos, or T_1_ neg and T_X_ neg), while a minority had positive or negative changes in repeated anti-ENA results (T_X_) when compared to their first anti-ENA sample (T_1_). Change neg = one or more autoantibodies became undetectable resulting in a negative anti-ENA test result; Change pos = alteration positive, one or more autoantibodies were detected that were not positive in the first anti-ENA test result; neg = negative; pos = positive.

The large majority (n = 3583, 86.1 %) of the patients with multiple anti-ENA tests had unchanged results as compared to the initial anti-ENA test result (Table 4). In more detail, consistent negative (T_1_ = neg, T_X_ = neg) results were observed in 1543 (37.1 %) patients, while 2040 (49.0 %) patients had consistent positive (T_1_ = pos, T_X_ = pos) results with no anti-ENA specificity alteration (Fig. 4).

Of the 4161 patients with repeated anti-ENA tests, 578 (13.9 %) patients had anti-ENA specificity alterations. In total, 335 (8.1 %) patients had 551 anti-ENA specificity alterations in which one or more of the detected autoantibodies in the first sample became undetectable in repeated analysis. In fact, 136 of these patients had a negative anti-ENA test after an initial positive anti-ENA result (T_1_ = pos, T_X_ = change neg). On the other hand, 243 (5.8 %) patients had 416 additional anti-ENA antibody specificities in follow-up testing. In 76 (1.8 %) patients anti-ENA test became positive after an initial negative result (T_1_ = neg, T_X_ = change pos), while in 167 (4.0 %) patients additional autoantibodies were detected after an initial positive anti-ENA result (T_1_ = pos, T_X_ = change pos).

## 4 Discussion

Repeated analysis of autoantibodies for AARD is commonly performed, though it is questioned if repeated anti-ENA testing is of any value. Additionally, both clinical requesting behavior and (laboratory) testing algorithms are not fixed in guidelines and there is a broad spectrum of assays with differences in performance characteristics potentially influencing test results. The present international multi-center study investigated the real-world efficacy of repeated anti-ENA testing according to local different AARD testing algorithms. We report three major findings.

First, it was observed that anti-ENA testing for AARD is increasingly performed, with the exception of the COVID-19 pandemic period. This decrease is due to the reduction in the number of autoantibody tests observed in all European countries during this period [18]. Additionally, more anti-ENA testing is in line with the growing prevalence of AARD due to the aging population, increased awareness and enhanced diagnosis, amongst others. Most anti-ENA requests and patients with a positive anti-ENA result, both for single and repeated assessments, involved female patients which is in line with the sex prevalence of autoimmune diseases [19].

Second, there was a substantial difference in negative versus positive anti-ENA test results in the total cohort when comparing MUMC to GUH and FSGDH. The rather low percentage of positive results in the MUMC cohort confirms previous findings [20]. This could be explained by the MUMC algorithm in which repeat ENA testing is not performed when repeat ANA testing demonstrated an identical pattern and titer, while GUH and FSGDH always perform ENA analysis after a positive ANA. Another explanation could be the lower pretest probability for the MUMC cohort. Due to a reduced sensitivity for anti-SSA antibodies by HEp-2 screening, clinicians are instructed to simultaneously request anti-ENA testing if anti-SSA antibodies might be expected. This is the case for requests from the dermatology department screening for cutaneous variants of SLE, as well as for requests from the neurology department. The latter is a referral center for small fiber neuropathy which is frequent in patients with Sjögren's syndrome. On the other hand, a higher prevalence of positive results could be expected in the FSGDH cohort due to the higher ANA titer, i.e. 1:160, required for reflex anti-ENA testing [21]. However, this does not hold for the GUH cohort, though GUH has many referrals for the Belgian Systemic Sclerosis Registry as well as for the workup of idiopathic inflammatory myopathies. Nevertheless, positive anti-ENA test prevalence of approximately 60–65 % for GUH and FSGDH was in line with previous findings of another tertiary center [22]. When assessing both the prevalence of the studied ENA specificities as well as the total number of positive specificities for all positive anti-ENA tests, comparable findings were found between the three centers which is in line with the prevalence of AARD and as shown in another literature study [10].

Third, by far the largest number of patients with repeated anti-ENA tests, which was more often performed if initially positive, had no altered result (86.1 %). The differences in the anti-ENA results have been mostly explained by the disappearance of one or more autoantibodies in follow-up testing (8.1 %), while in a minority of the population additional antibodies have been detected (5.8 %) as compared to the first anti-ENA test result. Importantly, only 76 (1.8 %) of the 4161 patients with repeated anti-ENA testing had a positive anti-ENA test after an initial negative anti-ENA result as in the study of Yeo et al. in which 53 patients (2.2 %) tested positive for anti-ENA after an initial negative finding from which only 5 patients had a new AARD [10]. Both clinical as well as (pre-)analytical reasons can explain the observed changes. Clinical reasons causing alterations might be initiation or discontinuation of AARD therapy, or early phase testing in developing AARD, among others. (Pre-) analytical reasons could be improper patient/blood sample identification, sample handling, Lot changes, processing delays and transportation errors [23]. Additionally, we have to consider that the inter-run variation for quantitative autoantibody assays is approximately 10–15 percent which could lead to different conclusion for values around the determined cut-off values [24].

This study has several limitations. The studied timeframe of each participating center differs. However, within these timeframes the used tests and algorithm were consistent in each of the centers excluding bias within each individual center. In addition, initial positive results within the timeframe of the current study might be preceded by a positive results prior to the chosen timeframe. Despite this study did not include clinical and treatment information this study did incorporate multiple centers with different local algorithms providing additional essential insights suggesting that repeated anti-ENA testing provides low diagnostic added value independently of the local algorithms in use [10,25]. Moreover, comparable concordance was observed in another single center study that did include clinical information with limited diagnostic yield. Also, the anti-ENA test results were studied as qualitative results without considering antibody levels. Hence, minor changes around the cutoff value could not be separated from major relevant changes. Nonetheless, interpretation of each result was in line with the manufacturer's instructions for use of each assay. Lastly, we did not include the specialties of the ordering physicians and whether the repeated and initial anti-ENA tests were ordered by the same physician or not.

## 5 Conclusion

In conclusion, in this retrospective study we demonstrated that repeated anti-ENA testing for AARD is common in clinical practice, independently of local laboratory testing algorithms. This reiteration seems to have limited added value since only 1.8 % of the patients demonstrated a positive anti-ENA test after an initial negative anti-ENA test. Our data do not support a proposal of a timeframe in which repeated testing is blocked, but at least suggest that repeated analysis should be discouraged and only be instigated by a change in clinical manifestations.

## CRediT authorship contribution statement

**Wim H.M. Vroemen:** Writing – original draft, Project administration, Formal analysis, Visualization, Methodology, Data curation, Writing – review & editing, Validation, Investigation, Conceptualization. **Maria Infantino:** Writing – review & editing, Data curation. **Mariangela Manfredi:** Writing – review & editing. **Joyce J.B.C. van Beers:** Writing – review & editing. **Carolien Bonroy:** Data curation, Writing – review & editing. **Jan G.M.C. Damoiseaux:** Resources, Writing – review & editing, Conceptualization, Supervision.

## Disclosures

No conflicts of interest to report regarding this manuscript.

## Sources of funding

This research did not receive any specific grant from funding agencies in the public, commercial, or not-for-profit sectors.

## Declaration of competing interest

The authors declare that they have no known competing financial interests or personal relationships that could have appeared to influence the work reported in this paper.

## Data Availability

The data of this study cannot be shared publicly due to privacy reasons of the study participants. Data will be shared upon reasonable request.
